# Supplementary material for: A Randomized Pilot Trial of Micronutrient Supplementation for Under-5 Children in an Urban Low-Cost Flat Community in Malaysia: A Framework for Community-Based Research Integration
Source: Int J Environ Res Public Health. 2022 Oct 25;19(21):13878. doi: 10.3390/ijerph192113878 (PMC9655965; doi:10.3390/ijerph192113878)
Supplement: Supplementary file 1 [file ijerph-19-13878-s001.zip › ijerph-1953796-supplementary.pdf]

SUPPLEMENTARY MATERIALS

S1: Summary measures (mean (SD)) of anthropometric, serum erythrocytic and iron storage profiles for each treatment arm (n=45)

S2: Patient information sheets and informed consent form (English Version)

S3: Patient information sheet and informed consent form (Malay Version)

S4: Case Report Form (CRF)

S5. Compliance Monitoring Log Sheet

Material S1. Summary measures (mean (SD)) of anthropometric, serum erythrocytic and iron storage profiles for each treatment arm (n=45)

Table S1. Summary measures (mean (SD)) of anthropometric, serum erythrocytic and iron storage profiles for each treatment arm (n=45)

**Variables**	**Control (n = 25) n (%)**		**Micronutrient (n = 20) n (%)**	
	**Pre (mean [SD])**	**Post (mean [SD])**	**Pre (mean [SD])**	**Post (mean [SD])**
Height Z score	- 1.29 [1.17]	- 1.12 [1.01]	- 0.85 [1.41]	- 0.94 [0.96]
Weight Z score	- 2.81 [1.73]	- 2.06 [1.59]	- 2.14 [1.83]	- 1.74 [1.76]
Body mass index Z score	- 3.30 [3.13]	- 2.21 [2.59]	- 2.48 [2.69]	- 1.70 [2.17]
Weight/ height Z score	- 3.18 [2.56]	-2.25 [2.66]	- 2.91 [2.66]	-1.97 [1.84]
Hemoglobin (g/L)	121.7 [12.8]	118.0 [7.4]	119.2 [11.6]	120.8 [9.9]
Mean corpuscular volume (fl)	72.8 [4.2]	74.4 [3.7]	72.9 [6.9]	76.9 [4.2]
Reticulocyte (%)	1.26 [0.39]	1.23[0.41]	1.10 [0.32]	1.44 [0.52]
Median reticulo-cyte hemoglobin (RET-HE) (pg)	29.2 [2.46]	30.3 [2.40]	28.5 [4.34]	30.2 [30.05]
Serum iron (µmol/L)	11.55 [4.28]	12.47 [4.81]	9.82 [4.12]	13.35 [8.91]

Serum ferritin (ug/L)	26.8 [17.6]	24.8 [14.6]	44.9 [59.3]	40.6 [24.9]
Serum transferrin (g/L)	2.50 [0.37]	2.61 [0.36]	2.60 [0.31]	2.46 [0.27]
Total iron binding capacity (TIBC) (umol/L)	62.7 [9.26]	65.40 [9.08]	65.3 [7.86]	61.7 [6.75]
Serum C reactive protein (mg/L)	0.86 [2.22]	0.38 [0.52]	1.45 [4.36]	1.07 [2.29]

Material S2. Patient information sheets and informed consent form (English Version)

PATIENT INFORMATION AND INFORMED CONSENT

Please read the following information carefully, and do not hesitate to discuss any questions you may have with your doctor.

Study Title: Micronutrient Supplementation for Child of Urban Poverty Iron Project (CUPIP)

Introduction:

Your child who is less than 5 years old is eligible to receive vitamin and mineral supplementation which might improve his /her overall growth and development. However, we do not know whether this supplementation will be effective for children in urban Malaysia.

What is the purpose of this study?

The purpose of the study is to determine whether 6 months of vitamin and mineral supplementation will affect the growth and iron level in the blood of young children.

What are the procedures to be followed?

Your child is eligible for the study if he/she is aged between 6 months and 5 years. You will be interviewed by a trained study coordinator to determine the dietary practices of your child and family. The height and weight of your child will be measured together with a 2 ml blood sample for iron status at the start and end of the study. Your child will receive a deworming treatment and will then be randomized to either receive vitamin and mineral supplementation for 6 months or receive no supplementation.

What will be benefits of the study?

Your child will receive deworming treatment and will be referred for additional medical care if his haemoglobin level (red blood cell) is very low. If he/she is randomized to the treatment group, he/she will receive vitamins and minerals to supplement daily nutrition. His/her growth will be monitored closely whether he/she receives vitamin and mineral supplements or not. Children who are randomized into the group which do not receive vitamins and minerals at the start will get these if their second blood results still show iron deficiency. Each child will be given RM20 for each blood sampling.

What are the possible drawbacks?

A small amount of blood will be collected at two time points. Your child might experience some discomfort during

the procedure. We will give a spray of stop-pain medication before blood test.

Can I refuse to take part in the study?

Yes. Your participation is completely voluntary and we would encourage you to take part in this study. However, if you do not want to be in the study, your decision will not in any way interfere with your child's baseline health.

Who should I contact if I have additional questions during the course of the study?

Study Coordinator: ______________. Contact: _____________________

INFORMED CONSENT

Study Title: Micronutrient Supplementation for Child of Urban Poverty Iron Project (CUPIP)

Patient's name: ____

Parent's name: ____

I confirm that I have been informed about the nature of the study. I have also received, read, and understood the above written information regarding this study. I consent to allow my child to participate in the study.

I may at any stage, without prejudice or penalty, withdraw my consent for my child's participation in this study.

**Name of parent:**	**Signature:**
**IC number:**	**Date:**
**Address:**	**Telephone number:**

Name of research assistant: __________________________ Signature: __________________

Date: ___________________

Name of Witness (if a translator is required): ___________________________

Signature: ___________________

Date: _______________

Material S3. Patient information sheets and informed consent form (Malay Version)

MAKLUMAT DAN PERSETUJUAN TERMAKLUM PESAKIT

Sila baca maklumat ini dengan teliti, jika terdapat kemusykilan atau pertanyaan, sila berhubung dan berbincang dengan doctor anda.

Tajuk kajian: Penambahan Mikronutrien Bagi Kanak-Kanak Dalam Golongan Miskin Bandar – Projek Zat Besi – Child of Urban Poverty Iron Project (CUPIP).

<u>Pengenalan:</u>
Anak anda yang berumur kurang daripada 5 tahun adalah layak untuk menerima suplemen zat vitamin dan mineral untuk membantu meningkatkan tumbesaran dan perkembangan fizikal. Walau bagaimanapun, kita tidak pasti sama ada suplemen ini berkesan untuk kanak-kanak di bandar Malaysia.

<u>Apakah tujuan kajian ini?</u>
Tujuan kajian ini dijalankan adalah untuk menentukan sama ada penambahan suplemen vitamin dan mineral selama 6 bulan berkesan untuk membantu meningkatkan pertumbuhan kanak-kanak dan tahap zat besi dalam darah.

<u>Apakah prosedur yang boleh diikuti?</u>
Anak anda layak mengikuti kajian ini jika anak anda berumur antara 6 bulan hingga 5 tahun. Anda akan ditemu-bual oleh pembantu kajian untuk menentukan amalan pemakanan (diet) anak dan keluarga anda. Ketinggian dan berat badan anak anda akan diukur dan sampel darah sebanyak 2ml (setengah sudu kecil) akan diambil untuk menentukan status kandungan zat besi dalam darah pada permulaan dan akhir kajian. Anak anda akan menerima rawatan ubat cacing dan kemudian akan dipilih secara rawak untuk menerima suplemen vitamin dan mineral selama 6 bulan atau tidak menerima suplemen.

<u>Apakah manfaat yang akan diperolehi melalui kajian ini?</u>
Anak anda akan menerima rawatan ubat cacing dan akan dirujuk untuk rawatan perubatan jika status haemoglobin (sel darah merah) terlalu rendah. Selain itu, jika anak anda terpilih untuk menerima suplemen, anak anda akan menerima vitamin dan mineral sebagai zat tambahan. Tumbesarannya akan dipantau dengan teliti sama ada dia menerima suplemen vitamin dan mineral. Bagi kanak-kanak yang tidak terpilih untuk menerima suplemen, pihak kami tetap akan memantau tumbesaran anak anda. Sekiranya keputusan anak anda menunjukkan kekurangan zat

besi semasa pemeriksaan darah kali kedua (selepas 6 bulan), anak anda akan diberi vitamin dan mineral sebagai rawatan. Setiap kanak-kanak akan diberi tunai sebanyak RM20 bagi setiap pengambilan darah.

<u>Apakah kelemahan yang mungkin dihadapi?</u>

2 sampel darah berjumlah 4 ml (jumlah bersamaan dengan satu sudu kecil) akan diambil daripada anak anda pada dua waktu yang berbeza. Anak anda akan mungkin berasa kurang selesa semasa proses pengambilan darah. Kami akan membantu dengan memberikan semburan ubat tahan sakit sebelum pengambilan darah.

<u>Bolehkah saya tidak bersetuju untuk mengambil bahagian dalam kajian ini?</u>

Ya, penyertaan anda adalah secara sukarela dan kami menggalakkan anda mengambil bahagian dalam kajian ini. Walau bagaimanapun, jika anda tidak bersetuju untuk mengambil bahagian dalam kajian ini, keputusan anda tidak akan mengganggu penjagaan yang akan anda / anak anda terima.

<u>Siapakah yang perlu saya hubungi jika saya mempunyai persoalan sepanjang kajian ini?</u>

Koordinator Kajian: ______________. **Nombor telefon: ____________________**

PERSETUJUAN TERMAKLUM

Tajuk Kajian : Penambahan Mikronutrien Bagi Kanak-Kanak Dalam Golongan Miskin Bandar – Projek Zat Besi – Child of Urban Poverty Iron Project (CUPIP).

Nama anak: ____

Nama ibu/ayah: ____

Saya mengesahkan saya telah dimaklumkan berkenaan kajian ini. Saya juga telah menerima, membaca, dan memahami maklumat bertulis di atas mengenai kajian ini. Saya bersetuju untuk membenarkan anak saya mengambil bahagian dalam kajian ini.

Saya boleh di mana-mana peringkat, tanpa prejudis atau penalti, menarik balik persetujuan saya untuk penyertaan anak saya dalam kajian ini.

**Nama ibu/ayah:**	**Tandatangan**
**No K/P:**	**Tarikh:**
**Alamat:**	**No telefon:**

Nama pembantu penyelidik: ______________________ **Tandatangan:** ________________

Tarikh: ________________

Nama saksi (jika penterjemah diperlukan): ______________________

Tandatangan: ________________

Tarikh: _______________

Material S4. Case Report Form (CRF)

MyKid# : ……………………………………………………………

STUDY ID:

To be completed by parent during first visit

Mother's name : ……

Mother's IC : ………………………………………………………………………… Age : ……………..

Address : ……

Contact Number : ………

Mother's height : ……………………..………..……………… Father's height : ……………………………..

Mother's details

1. Ethnicity

Malay	
Chinese	
Indian	
Others<br>Please specify :	

2. Occupation

Not working	
Self employed	
Paid employment	

3. Household income per month

< RM 3,000	
RM 3,000 – RM 6,500	
RM 6,500 – RM 16,000	
> RM 16,000	

4. Highest education qualification

UPSR	
PMR	
SPM	
Diploma	

5. Antenatal complications

Diabetes	
Hypertension	
Anemia	
Others<br>Please specify	
None	

6. Took iron containing supplements during pregnancy:

Yes	
No	

7. Took iron containing supplements currently

Yes	
No	

Currently pregnant: Y/N

8. Marital status

Single/never married	
Married	
Divorced	
Widowed	

Degree	
Master / Doctorate	

Baby's details

1. Gender

Male	
Female	

2. Period of gestation

37 – 38 weeks	
38 – 39 weeks	
39 – 40 weeks	
> 40 weeks	

3. Route of birth

Vaginal Delivery	
Caesarean section	

4. Feeding pattern (first 6 months of life)

Exclusively Breast Fed	
Mixed breast feed and formula feed *	
Exclusively formula fed *	

a) *If on formula milk

i) Brand

……………………….

ii) Amount (volume & frequency)

………………………………………..

9. Number of children: ……………………

5. Complementary food started (at least 2 meals within 24 hours):

Yes	
No	

6. Baby on any iron-containing supplement current

Yes	
No	

To be completed by parent during second visit

Name : ………

Mother's IC : ……………………………………………………………………………………………..

Contact Number : …………………………………………………………………………………………….

1. Mother taking iron containing supplements currently:

Yes	
No	

2. Baby's Current Feeding Pattern :

Exclusively Breast Fed	
Mixed breast feed and formula feed *	
Exclusively formula fed *	

a) * If on formula milk,

i. Brand:

………

ii. Amount (Volume and Frequency)

………

3. Complementary food started (at least 2 meals within 24 hours):

Yes	
No	

4. Baby on any iron containing supplements currently:

Yes	
No	

Material S5. Compliance Monitoring Log Sheet

STUDY ID:

PPR LEMBAH SUBANG CHILDREN'S OUTREACH PROJECT

Notes Schedule for Daily Supplement Taking (OCTOBER-NOVEMBER)

Name: Age: MyKid #: Phone number #:

DAILY NOTES

(Please fill in the date, signature and side effect issues if applicable)

	**Sunday**	**Monday**	**Tuesday**	**Wednesday**	**Thursday**	**Friday**	**Saturday**	**Volunteer Signature**
Week 1	Date: ___/___/___<br>Signature:<br>Complication:	Date: ___/___/___<br>Signature:<br>Complication:	Date: ___/___/___<br>Signature:<br>Complication:	Date: ___/___/___<br>Signature:<br>Complication:	Date: ___/___/___<br>Signature:<br>Complication:	Date: ___/___/___<br>Signature:<br>Complication:	Date: ___/___/___<br>Signature:<br>Complication:	Date: ___/___/___<br>Signature:<br>Complication:
Week 2	Date: ___/___/___<br>Signature:<br>Complication:	Date: ___/___/___<br>Signature:<br>Complication:	Date: ___/___/___<br>Signature:<br>Complication:	Date: ___/___/___<br>Signature:<br>Complication:	Date: ___/___/___<br>Signature:<br>Complication:	Date: ___/___/___<br>Signature:<br>Complication:	Date: ___/___/___<br>Signature:<br>Complication:	Date: ___/___/___<br>Signature:<br>Complication:
Week 3	Date: ___/___/___<br>Signature:<br>Complication:	Date: ___/___/___<br>Signature:<br>Complication:	Date: ___/___/___<br>Signature:<br>Complication:	Date: ___/___/___<br>Signature:<br>Complication:	Date: ___/___/___<br>Signature:<br>Complication:	Date: ___/___/___<br>Signature:<br>Complication:	Date: ___/___/___<br>Signature:<br>Complication:	Date: ___/___/___<br>Signature:<br>Complication:
Week 4	Date: ___/___/___<br>Signature:<br>Complication:	Date: ___/___/___<br>Signature:<br>Complication:	Date: ___/___/___<br>Signature:<br>Complication:	Date: ___/___/___<br>Signature:<br>Complication:	Date: ___/___/___<br>Signature:<br>Complication:	Date: ___/___/___<br>Signature:<br>Complication:	Date: ___/___/___<br>Signature:<br>Complication:	Date: ___/___/___<br>Signature:<br>Complication:

Greetings to parents/guardians,

I appreciate your willingness to allow your child to participate in this CUPIP Project for the sake of his future.

Introduction:

This CUPIP project is specially targeted at children aged five years and below. At this time, the formation of the brain and nervous system are rapidly developing. Iron and vitamin elements from supplementary food are essential for the development of children's brains. Lack of nutrients and vitamins causes stunted growth and learning problems in the future.

• Although the body's vitamin levels can be increased after children grow up, it is not able to restore their ability in the field of learning.

• By participating in the CUPIP project, your child is eligible to receive supplements containing 14 types of vitamins and minerals to help improve physical and brain growth and development. The nutritional effect of this supplement during the early years can be obtained throughout life, that is when children go to school until they reach adulthood.

• This project is indeed allowed to be carried out by the University of Malaya.

Important instructions

1. DO NOT SHARE THIS SUPPLEMENT WITH OTHER CHILDREN IN THE FAMILY OR WITH OTHER FAMILIES.
2. THIS SUPPLEMENT IS ONLY FOR CHILDREN WHO HAVE RECEIVED AN OVERALL HEALTH ASSESSMENT AND UNDERGONE A BLOOD TEST.
3. ONE PACK ONLY FOR ONE DAY, DO NOT EXCEED THE RECOMMENDED LIMIT. *Please keep the empty packaging. Content can be mixed into food or drink.*
